# Supplementary material for: Photosynthetic Linear Electron Flow Drives CO2 Assimilation in Maize Leaves
Source: Int J Mol Sci. 2021 May 5;22(9):4894. doi: 10.3390/ijms22094894 (PMC8124781; doi:10.3390/ijms22094894)
Supplement: Supplementary file 1 [file ijms-22-04894-s001.zip › ijms-1174299-supplementary.pdf]

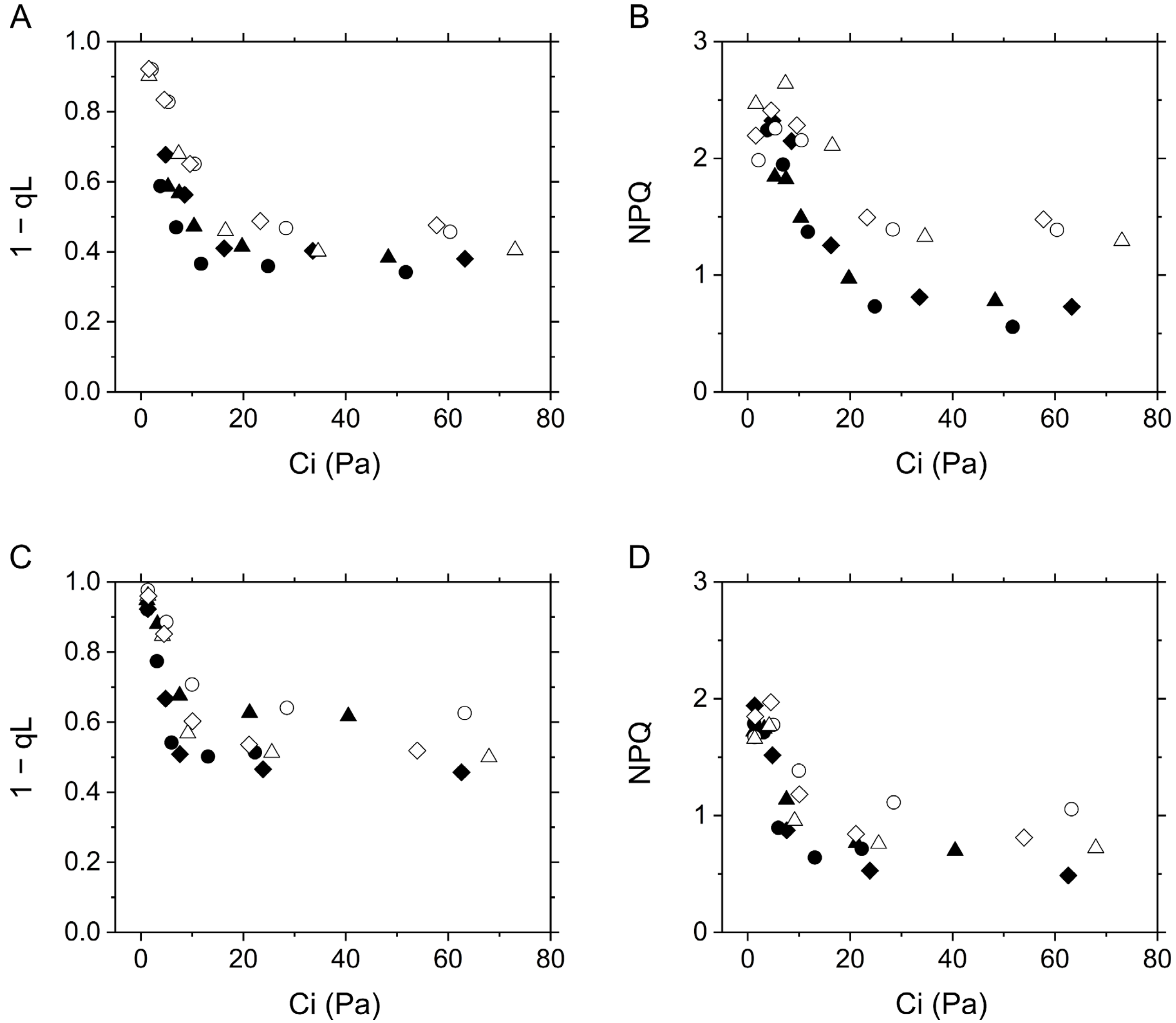

Supplemental Fig. S1. Inferred reduction level of plastoquinone (PQ) pool (1 − qL; A, C) and non-photochemical quenching (NPQ; B, D) at various intercellular CO_2 partial pressures (Ci) in the C_3 plant mustard (A, B) and the C_4 plant maize (C, D). Experiments were conducted independently three times as shown in different symbols (biological replicates) at 21 kPa (closed symbols) and 1 kPa O_2 (open symbols).

Supplemental Figure S1. Shimakawa and Miyake

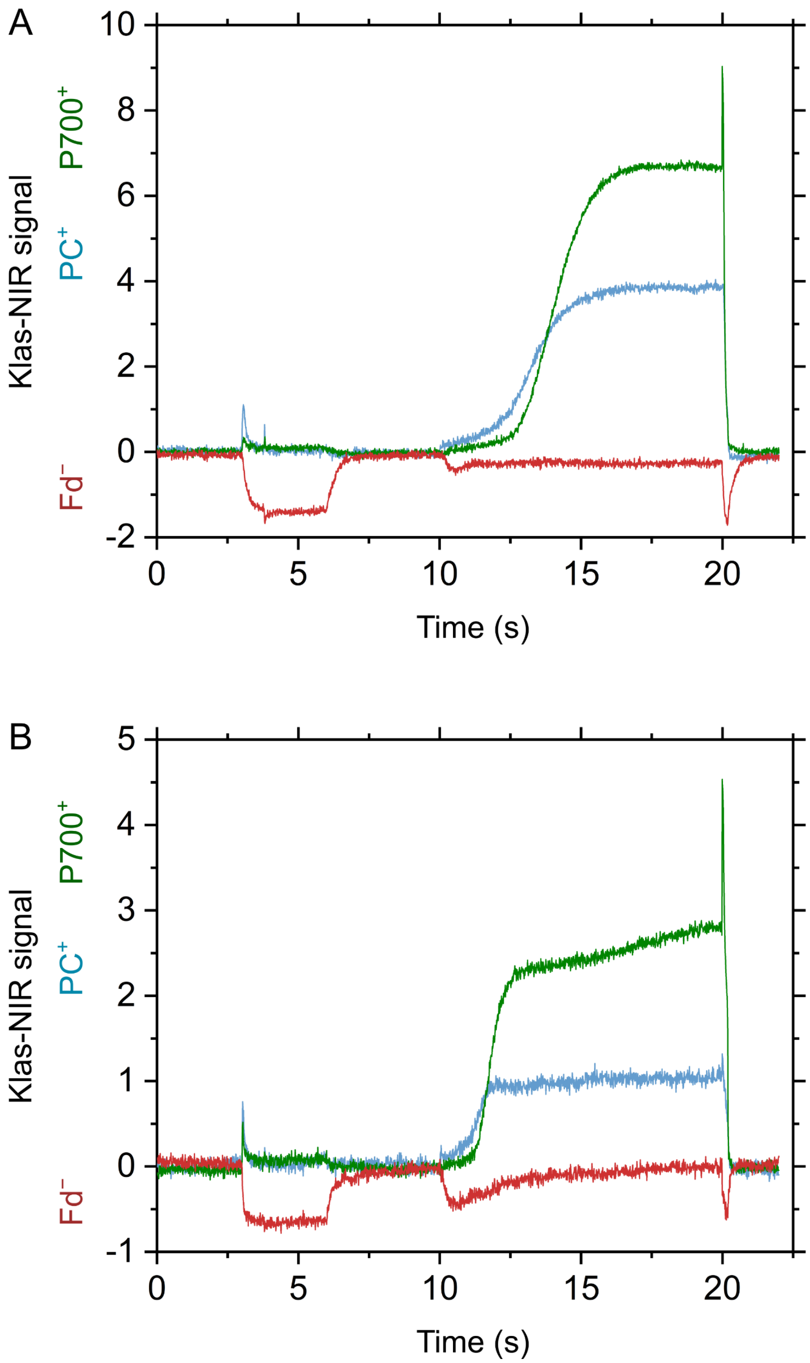

Supplemental Fig. S2. Responses of the redox states of P700^{+} (green), plastocyanin (PC^{+}, blue), and ferredoxin (Fd^{-}, red) to the illumination with red actinic light (3−6 s, 450 µmol photons m^{-2} s^{-1}) and far-red light (10−20 s) in the C_3 plant mustard (A) and the C_4 plant maize (B). Saturation flashes (10,000 µmol photons m^{-2} s^{-1}) were applied at 4 (30 ms) and 20 s (200 ms) respectively. The signal to Fd^{-} has negative values.

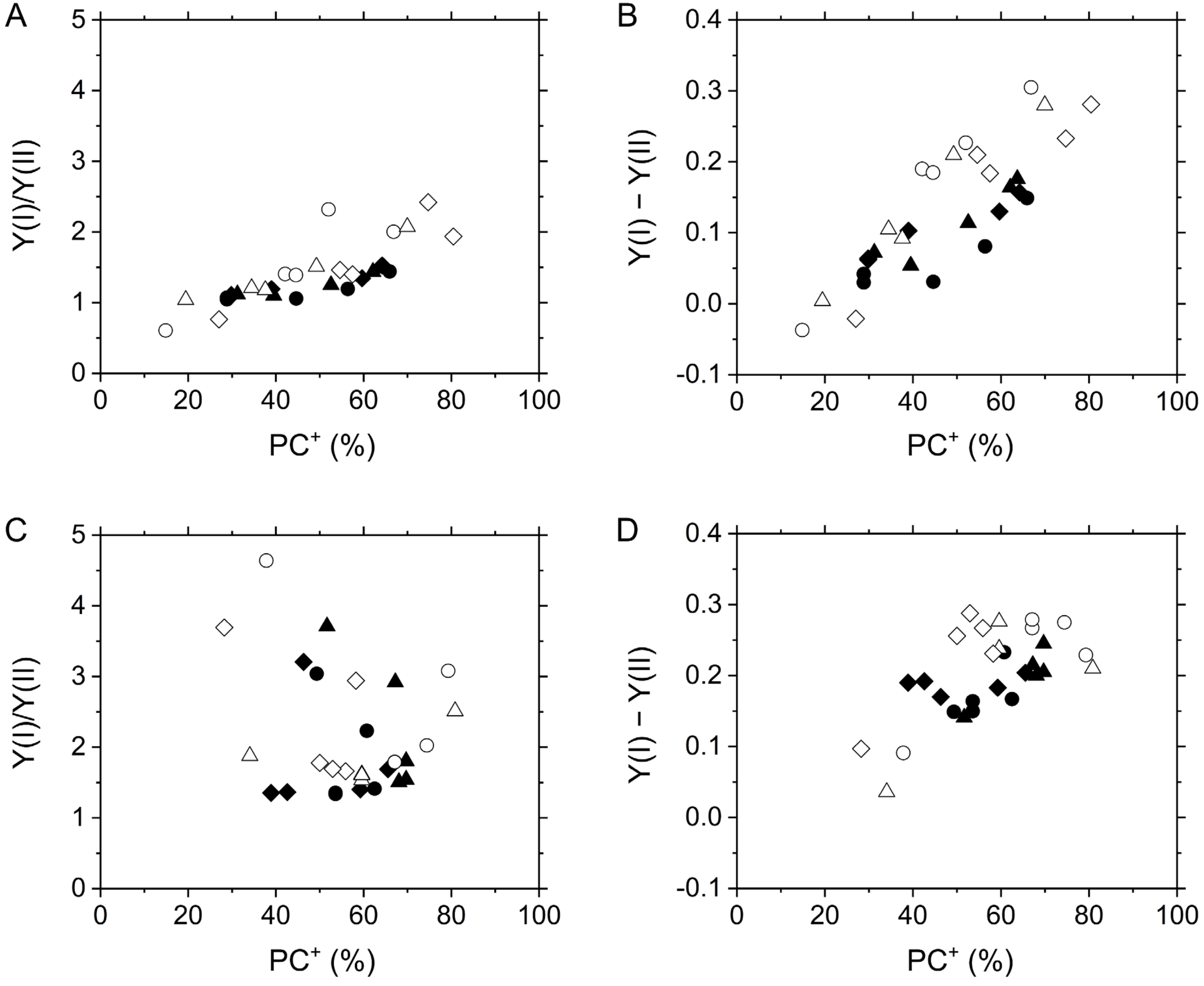

Supplemental Fig. S3. Relationships of the gap between effective quantum yield of PSI, Y(I), and of PSII, Y(II) with plastocyanin (PC) oxidation at various intercellular CO_2 partial pressures in the C_3 plant mustard (A, B) and the C_4 plant maize (C, D). Experiments were conducted independently three times as shown in different symbols (biological replicates) at 21 kPa (closed symbols) and 1 kPa O_2 (open symbols).

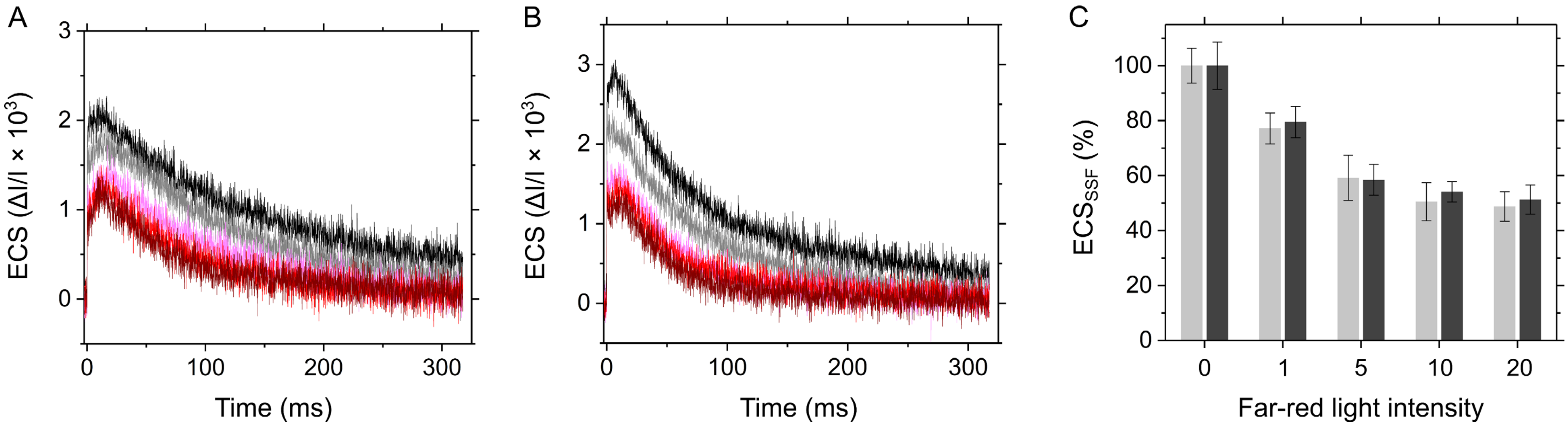

Supplemental Fig. S4. Electrochromic shift induced by a 5 µs-short saturation flash (ECS_{SSF}) during far-red light illumination in the C_3 plant sunflower (A) and the C_4 plant maize (B) grown in a field condition. Far-red light was provided at various intensities (0, black; 1, grey; 5, pink; 10, red; and the maximum 20, wine red; the values defined by the Walz software). (C) The flash-induced ECS changes normalized by the values without far-red light illumination as 100%. The data of sunflower (light grey) and maize (dark grey) are shown as the mean with the standard deviation (n = 3, biological replicates).
